# Progesterone produces antinociceptive and neuroprotective effects in rats with microinjected lysophosphatidic acid in the trigeminal nerve root

**DOI:** 10.1186/1744-8069-8-16

**Published:** 2012-03-19

**Authors:** Min Ji Kim, Hea Jung Shin, Kyoung Ae Won, Kui Ye Yang, Jin Sook Ju, Yoon Yub Park, Jae Sik Park, Yong Cheol Bae, Dong Kuk Ahn

**Affiliations:** 1 Department of Oral Physiology, School of Dentistry, Kyungpook National University, Daegu (700-422), South Korea; 2 Department of Oral Anatomy, School of Dentistry, Kyungpook National University, Daegu, South Korea; 3 Department of Physiology, School of Medicine, Catholic University of Daegu, Daegu, South Korea; 4 Department of Physiology, School of Medicine, Kyungpook National University, Daegu, South Korea

**Keywords:** Progesterone, Antinociception, Neuroprotection, Trigeminal neuralgia, LPA

## Abstract

**Background:**

In our present study, we studied the role of demyelination of the trigeminal nerve root in the development of prolonged nociceptive behavior in the trigeminal territory.

**Results:**

Under anesthesia, the Sprague-Dawley rats were mounted onto a stereotaxic frame and 3 μL of lysophosphatidic acid (LPA, 1 nmol) was injected into the trigeminal nerve root to produce demyelination. This treatment decreased the air-puff thresholds, persisted until postoperative day 130, and then returned to the preoperative levels 160 days after LPA injection. The LPA-treated rats also showed a significant hyper-responsiveness to pin-prick stimulation. We further investigated the antinociceptive and neuroprotective effects of progesterone in rats undergoing demyelination of the trigeminal nerve root. Progesterone (8, 16 mg/kg/day) was administered subcutaneously, beginning on the operative day, for five consecutive days in the LPA-treated rats. Treatment with progesterone produced significant early anti-allodynic effects and delayed prolonged anti-allodynic effects. The expression of protein zero (P0) and peripheral myelin protein 22 (PMP22) were significantly down-regulated in the trigeminal nerve root on postoperative day 5 following LPA injection. This down-regulation of the P0 and PMP22 levels was blocked by progesterone treatment.

**Conclusions:**

These results suggest that progesterone produces antinociceptive effects through neuroprotective action in animals with LPA-induced trigeminal neuropathic pain. Moreover, progesterone has potential utility as a novel therapy for trigeminal neuropathic pain relief at an appropriate managed dose and is therefore a possible future treatment strategy for improving the recovery from injury.

## Background

Progesterone is a female gonadal steroid hormone synthesized in the ovary that exerts a wide range of actions against its target tissues including the uterus, mammary glands and brain. There is however abundant evidence now that progesterone has functions that go beyond its role as a female sex hormone. Exogenous progesterone and its derivates have been shown to be a successful treatment for rat models of traumatic brain injury and stroke [1-4], and peripheral neuropathy [5-10]. Progesterone also reduces neuronal cell death [11] and attenuates neurological abnormalities after ischemia [12-14] and spinal cord injury [15]. These results suggest that progesterone or some of its metabolites can be successfully used to treat traumatic brain and spinal cord injury, as well as ischemic stroke. Therefore, the neurotrophic and neuroprotective effects of this hormone have attracted much attention because of their therapeutic promise.

The involvement of progesterone in the modulation of pain has been described in previous reports. In this previous study, the systemic administration of progesterone prevented the development of mechanical allodynia and reduced the painful responses to cold stimulation in animals subjected to a spinal cord hemisection [16]. In contrast, the intrathecal injection of ICI 182,780, a progesterone receptor antagonist, produced antinociceptive effects in another earlier study [17]. These controversial results suggest that although spinal progesterone receptors play an important role in neuropathic pain, the role of this hormone in pain modulation remains unclear.

Trigeminal neuralgia is a severe chronic pain syndrome characterized by brief but intense stabbing or electrical shock-like paroxysmal pain. In affected patients, adjacent arterial loops, tumors, or arteriovenous malformations have been shown to compress the trigeminal nerve root [18-20]. Previous studies from several decades ago proposed a causal relation between the presence of pain paroxysms and focal demyelination with compression of the trigeminal nerve root [18,19]. Hilton and colleagues [21] observed a focal loss of myelin and the demyelination of axons in the trigeminal nerve root by vascular compression in patient with trigeminal neuralgia. These results suggested that demyelination in the trigeminal nerve root plays an important role in the pathology of trigeminal neuralgia.

In our current study, we report prolonged nociceptive behavior in a rat model following focal demyelination produced by microinjection of lysophosphatidic acid (1-acyl-2-lyso-*sn*-glycero-3-phosphate; LPA) into the trigeminal nerve root. This procedure is simple and the treated animals manifest signs of chronic nociceptive behavior, including mechanical allodynia and hyperalgesia, in the trigeminal territory of the affected nerve. We also investigated the neuroprotective and antinociceptive effects of the systemic administration of progesterone in the LPA-treated rats. We administered progesterone subcutaneously beginning on the operative day for five consecutive days. After daily treatment with progesterone, we measured changes in the air-puff thresholds and we also monitored delayed anti-allodynic effects. Following the final administration of progesterone, changes in protein zero (P0) or peripheral myelin protein 22 (PMP 22) immunoreactivity were examined to evaluate its neuroprotective effects.

## Results

**Figure 1:**
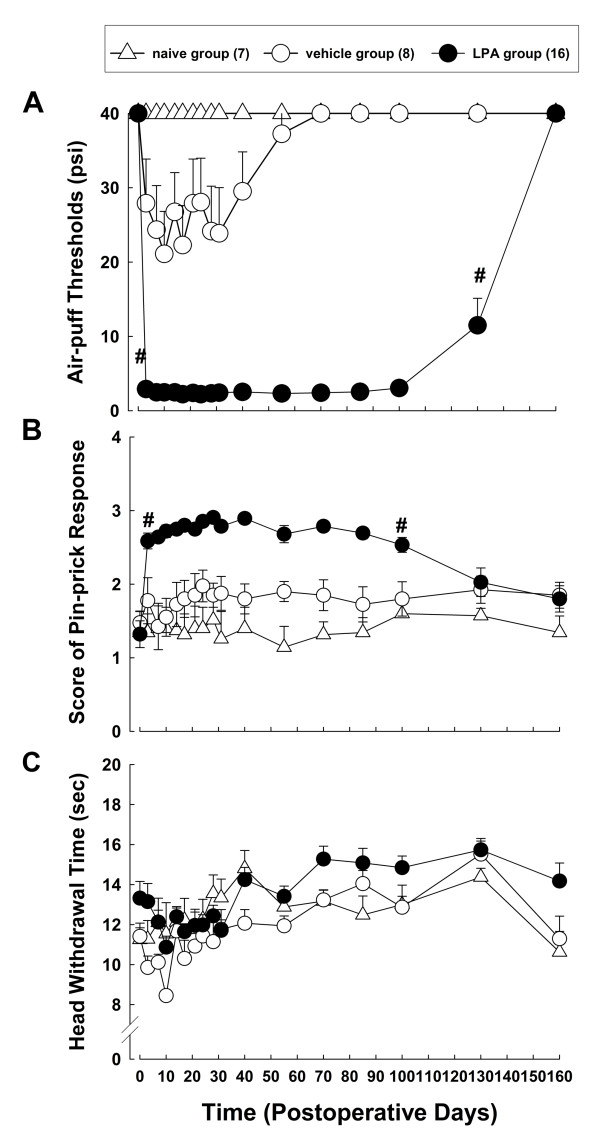
**Prolonged nociceptive behavior following the microinjection of LPA into the trigeminal nerve root of the rat**. (A) Time course analysis of changes in the air-puff thresholds. LPA-treated animals showed significant decreased air-puff thresholds on postoperative day 3. LPA-induced mechanical allodynia was maintained until postoperative day 130. (B) Time course analysis of changes in the pin-prick responses. LPA injection produced significant mechanical hyper-responsiveness to pin-prick stimulation compared with the vehicle-treated group. (C) Time course analysis of changes in the thermal nociception. *# P *< 0.05, vehicle- vs. LPA-treated group. All points between two identical symbols have the same level of significance.

Our present experiments demonstrated that LPA injected into the trigeminal nerve root of the rat produces prolonged nociceptive behavior in the trigeminal territory of the facial area (Figure 1). Naïve rats did not respond to a pressure of less than 40 psi. The vehicle-treated rats showed mild decreases in air-puff thresholds until postoperative day 30. However, the LPA-treated group showed significantly decreased air-puff thresholds as compared with the vehicle-treated group (F_(1, 22) _= 197.697, *P *< 0.001, Figure 1A). The mean air-puff threshold was decreased to 2.9 ± 0.3 psi at three days after microinjection of LPA and maintained until postoperative day 130. Air-puff thresholds recovered to their preoperative levels at 160 days after microinjection of LPA. We further observed mechanical hyperalgesia following LPA injection in our current analyses. The naïve animals did not produce any significant responses to pin-prick stimulation. Vehicle-treated animals showed increased responses to pin-prick stimulation, but this was not found to be statistically significant. However, LPA injection produced a significant hyper-responsiveness to pin-prick stimulation compared to the vehicle-treated group (F_(1, 22) _= 43.311, *P *< 0.001, Figure 1B). On postoperative day 3, the mean response to pin prick stimulation in the LPA-treated group (2.6 ± 0.1) was significantly higher than that in the vehicle-treated group (1.6 ± 0.3), which persisted until postoperative day 100. The head withdrawal latency times did not reveal any significant differences between the vehicle- and LPA-treated groups (Figure 1C). Body weight was also monitored after LPA injection. The mean body weight of the rats treated with LPA was not significantly different from that of the sham-treated group. All rats gained weight steadily after surgery and no significant differences were observed between the treatment groups (data not shown). These results suggest that the procedures we used did not produce surgical trauma or affect body weight.

**Figure 2:**
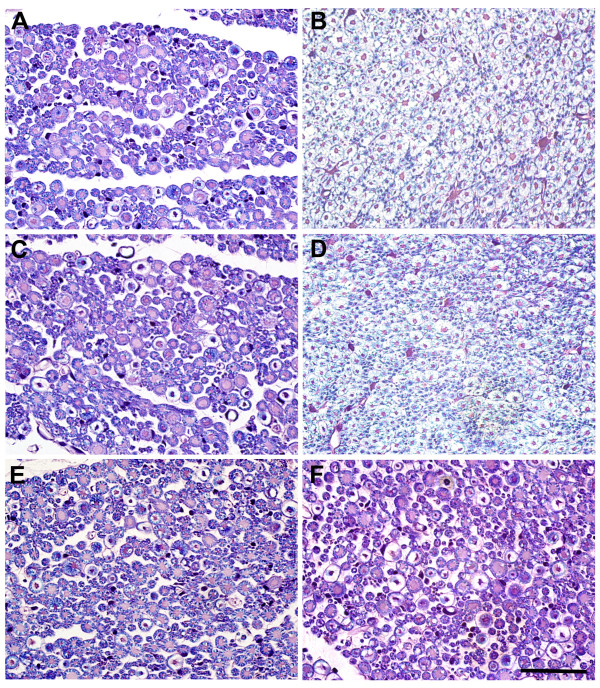
**The morphological changes in the trigeminal nerve root following microinjection of LPA**. On postoperative day 1 (A, B) and 14 (C, D), luxol fast blue stained light micrographs reveal severe demyelination of axonal portion of the trigeminal nerve root compared with vehicle-injected root, respectively (E, F). LPA-induced demyelination was recovered on postoperative day 160. Scale bar, 50 μm.

The morphological changes observed following LPA injection into the trigeminal nerve root are illustrated in Figure 2. The vehicle injection into the trigeminal nerve root did not affect the axonal morphology. At the light microscopic level, an LPA injection produced severe demyelination of the axonal portion of the trigeminal nerve root on postoperative day 1 (Figure 2A, B) and 14 (Figure 2C, D) compared to the vehicle-treated group, respectively. On postoperative day 160, morphological analysis showed that LPA-induced demyelination was recovered (Figure 2E, F).

**Figure 3:**
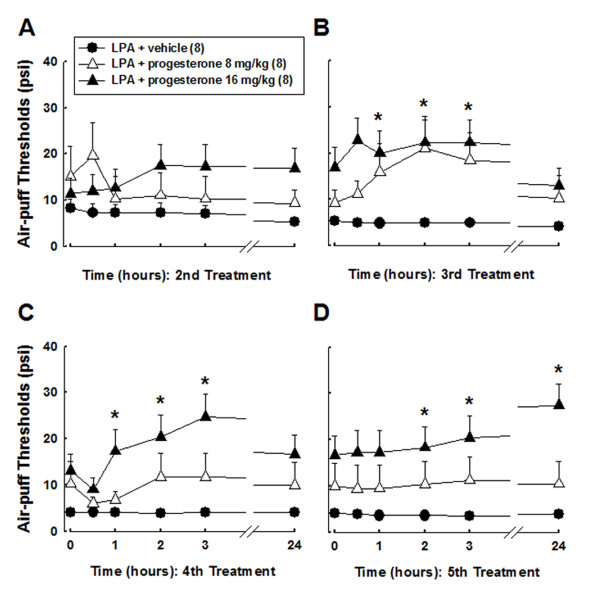
**The acute effects of daily treatments with progesterone (8 or 16 mg/kg/day) on mechanical allodynia in rats microinjected with LPA into the trigeminal nerve root**. Daily treatment with a low dose of progesterone (8 mg/kg/day) did not produce anti-allodynic effects. However, the administration of 16 mg of progesterone on postoperative days 3, 4, and 5 produced significant anti-allodynic effects. * *P *< 0.05, vehicle- vs. progesterone-treated group.

**Figure 4:**
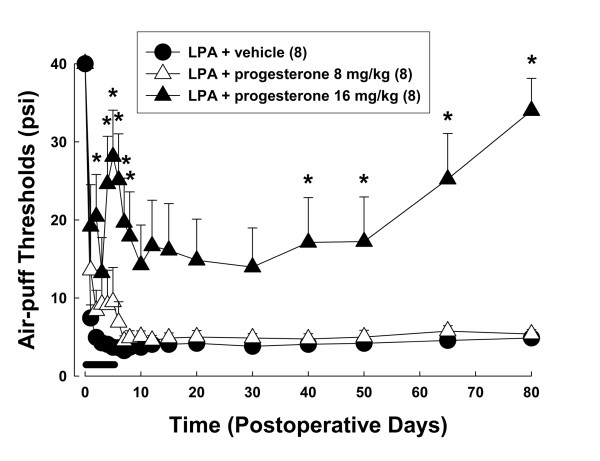
**Delayed effects of progesterone treatment (8 or 16 mg/kg/day) on mechanical allodynia produced by the microinjection of LPA into the trigeminal nerve root**. Progesterone-treated animals showed significant delayed anti-allodynic effects. The lines denote the period of five progesterone treatments. * *P *< 0.05, vehicle- vs. progesterone-treated group.

We observed antinociceptive effects following the administration of progesterone in rats with demyelination of the trigeminal nerve root. Progesterone (8, 16 mg/kg/day) was injected beginning on operative day for five consecutive days. The effects of daily treatment with progesterone upon the air-puff thresholds are illustrated in Figure 3. We did not monitor significant changes in air-puff thresholds following the first treatment with progesterone. Neither daily treatments with a low dose of progesterone (8 mg/kg/day) nor a high dose of progesterone (16 mg/kg/day) on postoperative day 2 produced anti-allodynic effects. However, after the administration of 16 mg of progesterone on postoperative days 3, 4, and 5, significant anti-allodynic effects could be observed (*P *< 0.05). Figure 4 illustrates the delayed effects of progesterone treatment upon the mechanical allodynia produced by LPA injection. Neither subcutaneous injection of vehicle nor a low dose of progesterone (8 mg/kg/day) affected mechanical allodynia. However, the subcutaneous injection of 16 mg of progesterone significantly reduced mechanical allodynia until four days after the final injection of progesterone. Moreover, progesterone-treated animals (16 mg/kg/day) showed delayed but significant anti-allodynic effects on postoperative day 40, compared to the vehicle-treated group (F_(1, 14) _= 14.532, *P *< 0.002). These delayed anti-allodynic effects persisted on postoperative day 80. To evaluate whether an antinociceptive dose of progesterone is associated with motor dysfunction, a rotarod test was performed after the subcutaneous administration of progesterone at 16 mg/kg/day. No effects on motor function were observed throughout the treatment period.

**Figure 5:**
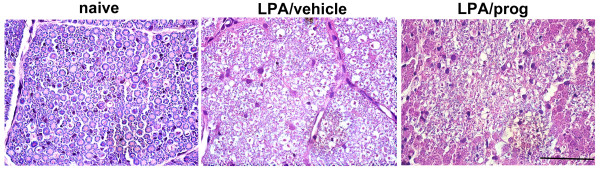
**The morphological changes in the trigeminal nerve root following treatment of progesterone**. Luxol fast blue stained light micrographs reveal the attenuation of demyelination of axonal portion of the trigeminal nerve root compared with vehicle-injected root after daily treatment of progesterone (16 mg/kg/day). Scale bar, 50 μm.

The effects of morphological changes observed following progesterone treatment (16 mg/kg/day) on LPA-induced demyelination in the trigeminal nerve root are illustrated in Figure 5. The vehicle injection into the trigeminal nerve root did not inhibit LPA-induced demyelination of the trigeminal root. However, daily treatment with progesterone reduced LPA-induced demyelination of the axonal portion of the trigeminal nerve root compared to the vehicle-treated group.

**Figure 6:**
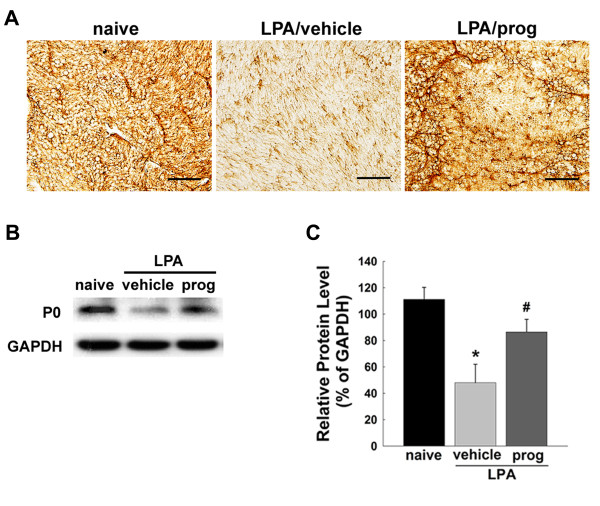
**Effects of progesterone treatment (16 mg/kg/day) upon P0 expression**. (A) Vehicle treatment did not affect the LPA-induced decreases in P0 expression in the trigeminal nerve root compared with naïve rats on postoperative day 5. However, decreased P0 expression, produced by LPA injection, was recovered following treatment with progesterone. (B) Western blot analysis confirmed that LPA injections cause a decrease in the 27 kDa band for P0, which was recovered by treatment with progesterone. GAPDH was used as a loading control. (C) Quantitative analyses of western blotting data. * *P *< 0.05, naive- vs. LPA/vehicle-treated group. # *P *< 0.05, LPA/vehicle-treated group vs. LPA/progesterone-treated group. Scale bar, 50 μm.

To assess the neuroprotective effects of progesterone against demyelination following LPA injection into the trigeminal nerve root, we examined P0 and PMP 22 expression using immunohistochemistry and western blotting analysis. Figure 6 illustrates the changes in P0 expression following LPA injection, showing significant down-regulation of this protein in the trigeminal nerve root on postoperative day 5 compared to the naïve animals. The down-regulation of P0 expression produced by LPA injection was blocked by progesterone treatment (16 mg/kg/day, Figure 6A). Western blot analysis also showed that treatment with progesterone significantly increased the P0 expression level in LPA-injected animals (*P *< 0.05, Figure 6B, C).

**Figure 7:**
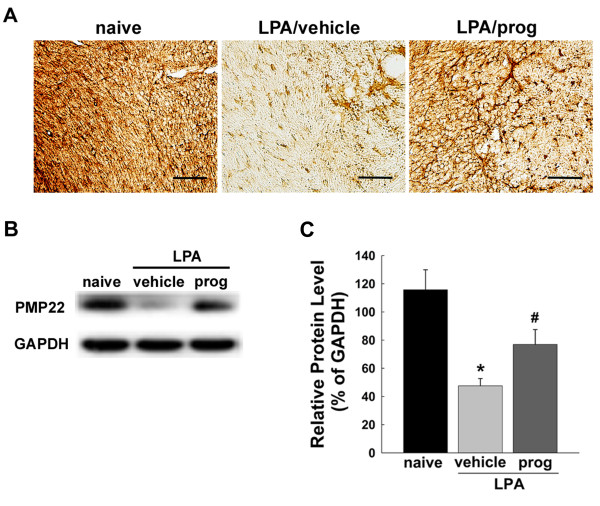
**Effects of progesterone treatment (16 mg/kg/day) upon PMP22 expression**. (A) Vehicle treatments did not affect the LPA-induced decreases in PMP22 expression in the trigeminal nerve root compared with naïve rats on postoperative day 5. However, decreased PMP22 expression, produced by LPA injection, was recovered following treatment with progesterone. (B) Western blot analysis also showed that LPA suppresses the 19 kDa band for PMP22, which was recovered by treatment with progesterone. GAPDH was used as a loading control. (C) Quantitative analyses of western blotting data. * *P *< 0.05, naive- vs. LPA/vehicle-treated group. # *P *< 0.05, LPA/vehicle-treated group vs. LAP/progesterone-treated group. Scale bar, 50 μm.

We obtained similar results when examining PMP 22 expression (Figure 7) which was found to be significantly down-regulated in the trigeminal nerve root on postoperative day 5 compared to the naïve animals. The down-regulation of PMP 22 expression produced by LPA injection was blocked by progesterone treatment (16 mg/kg/day, Figure 7A). This result was confirmed by western blot analysis (*P *< 0.05, Figure 7B, C).

## Discussion

Our present analyses demonstrate that the injection of LPA into the trigeminal nerve root produces severe demyelination and prolonged nociceptive behavior in rats. The subcutaneous administration of progesterone alleviates nociceptive behavioral responses and demyelination in a rat model involving microinjection of LPA into the trigeminal nerve root. These results suggest that progesterone produces antinociceptive effects through neuroprotective activity against trigeminal neuropathic pain produced by LPA-induced demyelination. Treatment with progesterone at an appropriate managed dose therefore has potential utility as a novel therapy for the relief of trigeminal neuropathic pain.

### Trigeminal neuropathic pain model

LPA is generated by enzymatic cleavage of stored glycophospholipids in the membranes of stimulated cells [22,23] and exhibits diverse biological activities during wound healing and tissue regeneration [24-26]. Inoue et al. [27] have recently demonstrated that the intrathecal administration of LPA produces demyelination causing neuropathic pain in mice. The participation of LPA-induced demyelination in the development of neuropathic pain is also supported by other LPA studies [28-30]. Moreover, LPA1 receptor knockout mice did not produce neuropathic pain resulting from LPA injection or demyelination following a partial sciatic nerve ligation [29]. A previous study has described a rat model in which LPA is injected into the trigeminal ganglion. These LPA-treated rats show severe demyelination in the trigeminal ganglion and significant nociceptive behavior. These results indicate that receptor-mediated LPA signaling plays a crucial role in the initiation of neuropathic pain --including the orofacial area.

In our present study, we have established a trigeminal neuropathic pain animal model involving LPA-induced demyelination in the trigeminal nerve root. The procedure is simple and the treated animals manifest signs of chronic nociceptive behavior, including mechanical allodynia and hyperalgesia, in the trigeminal territory of the affected nerve. LPA-treated rats also show sever demyelination in the trigeminal nerve root and this demyelination was recovered on postoperative day 160 in the present study. These results suggest that recovery of mechanical allodynia, produced by LPA injection, is caused by alleviation of demyelination in the trigeminal nerve root. Moreover, blockade of LPA receptor inhibited prolonged nociceptive behavior produced by LPA-induced demyelination in the trigeminal ganglion of rats [31]. These results support our findings that LPA-induced focal demyelination in the trigeminal nerve root produces severe and prolonged nociception. Interestingly, the etiology of this animal pain models may mimic that of trigeminal neuralgia because microvascular compression of the trigeminal nerve root was recognized as a cause of trigeminal neuralgia [19]. A focal loss of myelin of axons in the trigeminal nerve root were later further observed in a trigeminal neuralgia patient [21,32]. These results taken together with our findings may demonstrate a novel and significant animal model in which to study the trigeminal neuralgia.

### Neuroprotective effects of progesterone

It is well known that the progesterone synthesized by neurons and glial cells plays an important role in neuroprotection and myelination as a neurosteroid. A particularly noteworthy property of progesterone is that it not only provides strong neuroprotection but also promotes myelin formation, either during development or during the remyelination of axons in adults. We here observed neuroprotective effects of progesterone via the evaluation of P0 and PMP 22 levels in the trigeminal nerve root of LPA-treated rats. P0, a major protein component of peripheral nerve myelin, represents between 50-70% of the total protein and is a 28-kDa integral membrane glycoprotein which appears to be expressed by myelinating Schwann cells, but neither by central glia, nor by the non-myelinating Schwann cells that populate many peripheral nerves. PMP22 is a smaller 22-kDa glycoprotein that was first purified and characterized from bovine peripheral nerve myelin. It represents 2-5% of the total peripheral nerve myelin proteins.

The results of our present study demonstrate that LPA injection, which produces severe demyelination, significantly decreases the P0 and PMP 22 levels in the trigeminal nerve root. The down-regulation of P0 and PMP22 produced by LPA injection was blocked by progesterone treatment. These findings suggest that the systemic administration of progesterone attenuates the destruction of the myelin sheath caused by LPA injection. The neuroprotective effects of progesterone have been extensively studied in the rat spinal cord injury model or in the Wobbler mouse, a model of spontaneous motor neuron degeneration [33]. Progesterone also exerts protective effects on axonal pathology in experimental autoimmune encephalomyelitis, a multiple sclerosis animal model that manifests spinal cord demyelination [34]. The marked protective effects of progesterone on spinal motor neurons involve the up-regulation of brain-derived neurotrophic factor (BDNF), a key regulator of neuronal and glial functions [35]. Exogenous progesterone also enhances the concentrations of P0 and PMP22 proteins in co-cultures of dorsal root ganglion (DRG) neurons and Schwann cells [36] and stimulates the expression of their gene promoters in cultured rat Schwann cells [37]. These previous results suggest that progesterone plays an important role either in signaling the initiation of myelination or in enhancing the rate of myelin synthesis after lesion formation [38-41].

Progesterone is an excellent candidate molecule for neuroprotection and myelin repair, because it easily crosses the blood-brain barrier and exerts concerted beneficial influences on multiple processes [42]. The roles of the classical progesterone and non-classical GABA (A) receptors have been reported previously via observations in sciatic nerve and/or Schwann cells in the presence of these receptors [43]. On the basis of the observations in Schwann cell cultures, neuroactive steroids have been reported to stimulate P0 expression through classical progesterone receptor and PMP22 expression through GABA (A) receptor, respectively [44]. However, the direct interrelationship between antinociceptive effects and underlying mechanisms of neuroprotective action needs to be further explored.

### Antinociceptive effects of progesterone

Our present data demonstrate that a daily administration of progesterone produces a significant anti-allodynic effect. Interestingly, delayed and prolonged significant anti-allodynic effects were observed after the final administration of progesterone in our rat model. These results suggest that treatment with progesterone produces not only early anti-nociception but also delayed and prolonged anti-nociception. Accumulating behavioral evidence supports the antinociceptive effects of progesterone. The intrathecal administration of 3α, 5α-tetrahydroprogesterone (3α, 5α-THP) suppresses the thermal and mechanical nociceptive threshold in rats with neuropathic pain [45]. Molecular and biochemical investigations have also revealed an up-regulation of enzymatic pathways (cytochrome P450scc and 3α-hydroxysteroid oxidoreductase) leading to 3α,5α-THP biosynthesis in the dorsal horn [45-47] because cholesterol is converted to progesterone by cytochrome P450scc and 3β-hydroxysteroid dehydrogenase inside the steroidogenic mitochondria. Prolonged treatment with progesterone or its metabolites also show beneficial effects upon peripheral nerves at the neurophysiological, functional, and neuropathological levels in a model of diabetic neuropathy induced by an injection of streptozotocin in rats [6]. Progesterone may attenuate nociception and associated inflammatory responses via NK-1R (substance P receptor)-dependent pathways in vitro and in vivo studies [48].

Our present findings also demonstrate that daily treatments with progesterone produce significant anti-allodynic effects in the rats with LPA-induced demyelination without any motor impairment. Moreover, our current analyses further demonstrate that progesterone treatment significantly recovers the demyelination produced by LPA injection. Although our data do not indicate a direct interrelationship between a blockade of demyelination and antinociception, progesterone treatment seems to produce antinociception either by preventing demyelination or by promoting remyelination in our LPA-induced trigeminal neuropathic pain model. Hence, new strategies for developing treatments against trigeminal neuralgia like nociception, which simultaneously potentiate axonal regeneration, promote remyelination, or the recovery of nerve functions, are needed.

## Conclusions

In conclusion, the present analyses demonstrate that LPA injections into the trigeminal nerve root produce prolonged nociceptive behavior, including mechanical allodynia and hyperalgesia, in the trigeminal territory in our subject rats. The LPA-treated rats showed severe demyelination of the trigeminal nerve root. The subcutaneous administration of progesterone not only attenuated mechanical allodynia but also recovered demyelination or promoted remyelination in these animals. These results suggest that progesterone produces antinociceptive effects through neuroprotective action. Hence, progesterone has potential utility as a novel therapy for trigeminal neuralgia-like pain relief at an appropriate managed dose and is therefore a possible future treatment strategy for improving the recovery from injury.

## Methods

### Animals

Experiments were carried out using male Sprague-Dawley rats weighing between 180 and 210 g. The animals were maintained in a temperature-controlled room (23 ± 1°C) with a 12/12 hour light-dark cycle. All procedures involving the use of the animals were approved by the Institutional Animal Care and Use Committee of the School of Dentistry, Kyungpook National University, and were carried out in accordance with the ethical guidelines for the investigation of experimental pain in conscious animals proposed by the International Association for the Study of Pain.

### Development of animal model by LPA injection into the trigeminal nerve root

Surgical procedures were performed under pentobarbital sodium (40 mg/kg, i.p.) anesthesia. Anesthetized rats were mounted on a stereotaxic frame (Model 1404; David Kopf Instruments, Tujunga, CA) for LPA injection into the left trigeminal nerve root. A 3 μL volume of LPA (1 nmol) was injected into the trigeminal nerve root through a glass micropipette (tip diameter 50 μm) connected to a 10 μL Hamilton syringe (31 gauge) for 10 sec. The site of injection was 7.2 mm posterior to the bregma, 2.8 mm lateral from the midline, and 9.6 mm ventral from the surface of the skull. Ten minutes later, the glass micropipette was withdrawn, the wound was sutured, and a local anesthetic ointment was applied. After each experiment, the rats were intracardially perfused with 100 ml of heparinized normal saline followed by 500 ml of freshly prepared fixative solution under deep anesthesia with sodium pentobarbital (80 mg/kg, i.p.). After perfusion, the LPA injection site was identified using a stereoscope. Only data obtained from rats with injection sites clearly within the trigeminal nerve root were used in the behavioral analysis. LPA was purchased from Biomol and dissolved in 0.25% methanol in saline.

### General procedures for behavioral testing

Rats were randomly assigned to one of three groups. In the LPA-treated group (n = 16), the animals received an LPA injection into the trigeminal nerve root. In the control group, the rats received a vehicle injection (n = 8). Behavioral responses were also examined in a naïve group (n = 7) that did not receive any surgery or treatments. All behavioral tests were conducted between 0700 and 1800 hours. Rats were tested 3 days before and at 3, 7, 10, 14, 17, 21, 24, 30, 40, 55, 70, 85, 100, 130 and 160 days after the LPA injection. We measured the body weights of the animals after behavioral monitoring and returned them to their cages. All behavioral responses were measured by an experimenter in a blind fashion.

#### Evaluation of orofacial mechanical allodynia

To conduct behavioral observations, each rat was placed in a customized observation cage which was then placed in a darkened and noise-free room. The animals were then acclimated for at least 30 min. Withdrawal behavior, produced by 10 successive trials of constant air-puff pressure (4 sec duration, 10 sec intervals), was then examined in freely moving rats, as described previously [31,49,50]. The intensity and intervals of the air-puff pressure were controlled with a pneumatic pump module (BH2 system; Harvard Apparatus, Holliston, MA). The air-puffs were applied through a 26-gauge metal tube (length, 10 cm) located 1 cm from the skin at a 90° angle. Thresholds were determined through the air-puff pressure at which each rat responded in 50% of the trials. The cut-off pressure for the air-puffs was 40 psi. The naïve rats did not respond to a pressure of less than 40 psi. A significant decrease in air-puff thresholds compared with the pre-operative values was defined as mechanical allodynia.

#### Evaluation of orofacial mechanical hyperalgesia

Mechanical hyperalgesia was assayed using a pin-prick test following the same strict rule of habituation and testing environments for von Frey testing, as described previously [31,50]. Briefly, the rats were left to adapt to the observation cage for 30 min prior to the actual stimulation. During this period, an experimenter reached into the cage every 30 sec to touch the walls of the cage with a plastic rod, similar to those used to induce pain, as previously described [51]. After the rats became accustomed to the reaching movements, a series of mechanical stimulations were conducted. A blunt acupuncture needle (Needle No. 3, Serine, Japan) was applied until the needle was slightly bent (the skin was dimpled but not penetrated). The rat response to the stimuli was scored on an ordinal scale [31,49,50] as follows: no response, 0; detection, 1; detection and withdrawal, 2; detection, withdrawal, and escape or attacking movements, 3; as for response 3, but with prolonged facial grooming (> 3 strokes), 4.

#### Evaluation of orofacial thermal hyperalgesia

Each rat was placed in a customized cylinder-type acrylic rodent restrainer (height 40 mm-60 mm, length 70-120 mm) for the evaluation of thermal hyperalgesia. The cages had a hole in the top so that the head could receive thermal stimulation and produce a head withdrawal action. Each cage was placed in a darkened and noise-free room and the animals were habituated for at least 30 min prior to the start of the experiment. After radiant heat was applied, the head withdrawal time was determined as described in previous studies [31,52]. The application of heat stimuli was performed using an infrared thermal stimulator (Infrared Diode Laser, LVI-808-10; LVI tech, Seoul, Korea). The power and current of the thermal stimulator were adjusted to 11 W and 18.1 A, respectively. This intensity of the thermal stimuli produced stable latencies of approximately 12 sec for a 10 cm distance from the heat source to the vibrissa pad. Each rat received two stimuli and the inter-stimulus interval for each trial was at least 5 min. A cut-off time of 20 seconds was used in these experiments to prevent possible tissue damage.

### Morphological investigations

Under deep anesthesia with sodium pentobarbital (80 mg/kg, i.p.), rats (n = 6) were intracardially perfused with 100 ml of heparinized normal saline, followed by 500 ml of 4% paraformaldehyde in 0.1 M phosphate buffer (PB, pH 7.4) on postoperative day 14 or 160. After perfusion, the LPA injection site was identified. The trigeminal nerve root was then removed, postfixed in the same fixatives overnight, and then embedded in paraffin and sectioned (4 μm thick) for light microscopy. Sections were stained with luxol fast blue, which identifies the myelin sheaths of the axons. The histology of each section was examined under a light microscope.

### Evaluation of antinociceptive and neuroprotective effects of progesterone

To investigate the antinociceptive effects of progesterone, we administered this hormone subcutaneously in LPA-treated animals. Progesterone (8, 16 mg/kg/day) was injected beginning on operative day for five consecutive days. This concentration of progesterone was shown previously to recover behavioral performance in rats after medial frontal cortical contusions [53]. After these daily treatments, changes in the air-puff thresholds were measured at 30, 60, 120, 180 min, and 24 hours. We also tested for any delayed anti-allodynic effects of progesterone after the fifth treatment. To investigate neuroprotective effects of progesterone following the final administration of progesterone at 16 mg/kg/day, changes in P0 or PMP 22 immunoreactivity were examined. Progesterone was purchased by Sigma and dissolved in 200 μl sesame oil.

### Immunohistochemical staining

After the fifth treatment with progesterone, some of the rats (n = 5 per group) were perfused through the ascending aorta with 0.9% saline, followed by 4% paraformaldehyde in 0.1 M PB. The trigeminal nerve roots were then dissected out, postfixed in the same fixative at 4°C overnight, and then replaced with 30% sucrose in 0.1 M PB overnight. Transverse frozen sections (free-floating, 18 μm) were performed by a cryostat and processed for immunohistochemistry. All sections were blocked with 5% goat serum in PBS containing 0.2% Triton X-100 for 1 h at room temperature. Subsequently, they were incubated overnight at 4°C with rabbit polyclonal anti-P0 (1:500; Abcam, Cambridge, UK) or rabbit polyclonal anti-PMP22 (1:500; Abcam). The sections were then incubated with horseradish peroxidase (HRP)-conjugated anti-mouse and anti-rabbit antibody (Vector Laboratories, Burlingame, CA) for 2 h at room temperature. The sections were next covered with DAB solution (Vector Laboratories) for 10 min and observed under a light microscope (Axioplan; Zeiss, Oberkochen, Germany).

### Western blotting

Rats (n = 5 per group) were sacrificed by decapitation after their fifth treatment with progesterone. The trigeminal nerve root including the injection site was immediately removed and quickly frozen in liquid nitrogen. Samples were sonicated with Biorupture (Cosmo Bio., Tokyo, Japan) in a lysis buffer containing protease and a phosphatase inhibitor cocktail (Thermo Scientific, Rockford, IL). Protein concentrations in the samples were measured using a fluorometer (Invitrogen, Carlsbad, CA). For western blotting, total proteins (30 μg) were separated in a 4-12% gradient NuPAGE Novex Bis-Tris gel (Invitrogen) and transferred onto a PVDF membrane using an iBlot Dry blotting system (Invitrogen). The membranes were then blocked with 5% non-fat milk in TBS with 0.1% Tween 20 for 1 h at room temperature and then incubated with P0 (1:2000, Abcam) or PMP 22 (1:2000, Abcam) at 4°C overnight. The blots were subsequently incubated with goat anti-rabbit horseradish peroxidase for 1 h at room temperature. Membranes were developed using the SuperSignal West Femto substrate (Pierce, Rockford, IL), and exposed to X-ray film. We used the ImageJ analysis system (NIH, Bethesda, MD) to quantify specific bands.

### Rotarod test

Changes in motor performance after the subcutaneous administration of progesterone were measured using a rotarod (Ugo Basile, Comerio-Varese, Italy), as described previously [54,55]. The rotarod speed was set at 12 rpm with a maximum time spent on the rod set at 180 sec. The rats received two or three training trials on two separate days prior to testing for acclimatization. On the testing day, the resting response was examined. After the subcutaneous administration of progesterone (16 mg/kg/day), the time course of the motor performance was examined.

### Statistics

Differences between groups were compared using analysis of repeated measures analysis of variance (ANOVA) followed by LSD post hoc analysis. Comparisons between two means were performed using the Student's *T*-test. In all statistical comparisons, *P *< 0.05 was used as the criterion for statistical significance. All data are presented as the mean ± SEM.

## Abbreviations

LPA: Lysophosphatidic acid; P0: Protein zero; PMP22: Peripheral myelin protein 22; BDNF: Brain-derived neurotrophic factor; DRG: Dorsal root ganglion; 3α 5α-THP: 3α, 5α-tetrahydroprogesterone; PB: Phosphate buffer; PBS: Phosphate-buffered saline; ANOVA: Analysis of variance.

## Competing interests

The authors declare that they have no competing interests.

## Authors' contributions

MJK and HJS carried out the experiment and drafted the manuscript. KAW, KYY, JSJ, YYP, and JSP participated in the design of the study. YCB help conceive of the study, and participated in its design. DKA coordinated and supervised the experiments, analyzed the data and wrote the manuscript. All authors read and approved the final manuscript.
